# miR-193a-3p Mediates Placenta Accreta Spectrum Development by Targeting EFNB2 via Epithelial-Mesenchymal Transition Pathway Under Decidua Defect Conditions

**DOI:** 10.3389/fmolb.2020.613802

**Published:** 2021-01-13

**Authors:** Na Li, Rui Hou, Tian Yang, Caixia Liu, Jun Wei

**Affiliations:** ^1^Department of Obstetrics and Gynecology, Shengjing Hospital of China Medical University, Shenyang, China; ^2^Key Laboratory of Maternal-Fetal Medicine of Liaoning Province, Key Laboratory of Obstetrics and Gynecology of Higher Education of Liaoning Province, Benxi, China

**Keywords:** miR-193a-3p, EFNB2, epithelial-mesenchymal transition, decidua defect, placenta accreta spectrum

## Abstract

**Objective:** To clarify the role of microRNA-193a-3p (miR-193a-3p) in the pathogenesis of placenta accreta spectrum.

**Methods:** The placental tissue expression levels of miR-193a-3p and Ephrin-B2 (EFNB2) were compared between a placenta accreta spectrum group and a control group. Transwell migration and invasion assays were used to verify the effect of miR-193a-3p and EFNB2 on HTR-8/SVneo cells cultured in human endometrial stromal cell (hESC)-conditioned medium. Epithelial-mesenchymal transition (EMT)-related proteins were examined by western blotting to establish whether the EMT pathway was altered in placenta accreta spectrum. To determine whether EFNB2 is a target gene of miR-193a-3p, luciferase activity assays were performed.

**Results:** miR-193a-3p was upregulated but EFNB2 downregulated in the placenta accreta spectrum group and EFNB2 was a direct target of miR-193a-3p. Overexpression or inhibition of miR-193a-3p revealed that miR-193a-3p promoted the migration and invasion of HTR-8/SVneo cells cultured in hESC-conditioned medium. Furthermore, EMT was induced, as shown by increased N-cadherin, vimentin, MMP2, and MMP9 and decreased E-cadherin in the placenta accreta spectrum group and in HTR-8/SVneo cells transfected with miR-193a-3p mimics or si-EFNB2. The negative effect of miR-193a-3p inhibitor was reversed by co-transfection with si-EFNB2 in function studies and in analyses of EMT-related proteins *in vitro*.

**Conclusion:** miR-193a-3p which upregulated in placenta accreta spectrum group increases HTR-8/SVneo cell migration and invasion by targeting EFNB2 via the EMT pathway under decidua defect conditions to lead to placenta accreta spectrum.

## Introduction

Placenta accreta spectrum (PAS) refers to a pathological condition of placentation in which the placental chorionic villi adhere abnormally to or invade the uterine wall (Jauniaux and Jurkovic, 2012; Jauniaux et al., 2018). The prevalence of PAS has increased in recent years and it now affects 2–90 per 10,000 births (Thurn et al., 2016). However, its prevalence can be as high as 1 in 272 in the United States in women with birth-related hospital discharge diagnosis (Mogos et al., 2016). Differences in study populations and diagnostic criteria may account for this wide range in prevalence. Although PAS is a major cause of maternal morbidity and mortality, few studies have explored the underlying molecular mechanisms. Elucidation of these mechanisms would provide diagnostic and prognostic markers and potential targets for therapeutic strategies targeting PAS. Although the decidua defect is the primary effect, PAS is also thought to be associated with trophoblast over-invasion and abnormal angiogenesis (Jauniaux and Jurkovic, 2012).

MicroRNAs (miRNAs) are small non-coding RNA molecules (~22 nucleotides long) that suppress gene expression by binding to the 3′ end of the untranslated region (3′-UTR) of target mRNAs (Bartel, 2004). miRNAs play a critical role in placental development (Farrokhnia et al., 2014; Vaiman, 2017; Hayder et al., 2018) and, although a small number of studies have examined miRNA expression profiles and miRNA function in severe preeclampsia, early pregnancy loss, and fetal growth restriction (Choi et al., 2013; Higashijima et al., 2013; Yang et al., 2015; Gunel et al., 2017; Hosseini et al., 2018), fewer studies have sought to identify the involvement of miRNA in PAS. miR-34a, miR-29a/b/c, and miR-125a are significantly downregulated in PAS (Umemura et al., 2013; Gu et al., 2016, 2019) and might contribute to the development of PAS. Based on our previous miRNA expression profiles comparing PAS and non-PAS groups, we determined that miR-193a-3p, initially identified by Lagos-Quintana in 2003 (Lagos-Quintana et al., 2003), had higher expression levels in placental tissues in the PAS group (Yang et al., 2020). Bioinformatic screening to predict the target sites for miR-193a-3p was performed by using Tarbase (Karagkouni et al., 2018) and indicated that Ephrin-B2 (EFNB2) was a target gene of miR-193a-3p.

EFNB2 is a transmembrane ligand for Eph receptors belonging to the largest family of receptor tyrosine kinases, which are expressed spatially and developmentally during human placentation (Chennakesava et al., 2006). In preeclampsia, EFNB2 can inhibit endothelial progenitor cell-mediated vasculogenesis *in vitro* (Liu et al., 2015). However, only a few studies have investigated the role of EFNB2 in trophoblast invasion. Furthermore, EFNB2 has a critical role in maintenance of epithelial differentiation (Loffredo et al., 2017) and miR-193a-3p may be an epithelial-mesenchymal transition (EMT) related biomarker in colorectal cancer (Lin et al., 2017).

EMT refers to the process by which epithelial cells undergo a number of biochemical changes to acquire the mesenchymal cell phenotype (Kalluri and Weinberg, 2009). EMT plays a critical role in extravillous trophoblast differentiation and placental development, which might be involved in pathological pregnancies (Kokkinos et al., 2010; Davies et al., 2016). Moreover, extravillous trophoblasts from PAS show more mesenchymal characteristics and E-cadherin loss, which are consistent with a more invasive phenotype (Duzyj et al., 2015; DaSilva-Arnold et al., 2018). However, the precise EMT-associated mechanisms in trophoblast invasion remain unclear.

In this study, we determined the expression of miR-193a-3p in placental tissues in PAS patients and investigated the effects of miR-193a-3p on cell migration and invasion *in vitro*. Based on reporter assays, we determined that miR-193a-3p directly targets the EFNB2 gene and acts via the EMT pathway in HTR-8/SVneo cells under decidua defect conditions.

## Methods

### Study Population and Specimen Collection

Women from Shengjing Hospital, China Medical University, Shenyang, Liaoning, were recruited if they had been diagnosed with placenta previa based on ultrasonography, had a history of at least one previous cesarean delivery, and had a pregnancy terminated by cesarean delivery. Patients were excluded if any of the following criteria were met: multiple pregnancy, fetal anomalies, pre-term premature rupture of membranes, or infection and complications associated with any other obstetric diseases, such as thyroid dysfunction, hypertension, and gestational diabetes. Informed consent was obtained from all participants included in the study.

The participants were divided into either a PAS group (*n* = 20) or a control group (*n* = 20) according to a placenta combined with tightly connected uterus pathology examination after cesarean delivery. And the PAS was diagnosed by pathologists according to the villi whether adhere or invade the uterine wall with the depth of invasion. Furthermore, the PAS in our study included the placenta creta (in which the villi simply adhere to the myometrium), placenta increta (in which the villi invade the myometrium) and placenta percreta (in which the villi invade the full thickness of the myometrium).

The study was approved by Shengjing Hospital and China Medical University.

### Cell Lines, Cell Culture, and Transfection

The immortalized human trophoblast cell line HTR-8/SVneo was cultured in RPMI 1640 medium (cat. no. 01-100-1A; Bioind, Beit Haemek, Israel) supplemented with 10% fetal bovine serum (FBS; cat. no. 04-001-1A; Bioind) (termed complete RPMI) at 37°C in an atmosphere containing 5% carbon dioxide. For transient transfections, 1 × 10^6^ HTR-8/SVneo cells were plated into 6-well-plates in complete RPMI. When the cells reached 60–70% confluency, miR-193a-3p mimics, miR-193a-3p inhibitors, si-EFNB2, or their negative controls (GenePharma, Suzhou, China, concentration was 20 uM) were transfected into the cells using Lipofectamine 3000 reagent according to the manufacturer's instructions (cat. no. L3000015; Invitrogen, Carlsbad, CA, USA). At 6 h after transfection, the culture medium was changed to human endometrial stromal cell (hESC)-conditioned medium supplemented with 10% FBS (termed complete hESC conditioned medium). Cells were harvested for subsequent assays at 48 h after transfection.

For luciferase assay, 1 × 10^5^ HTR-8/SVneo cells were seeded in 24-well-plates in complete RPMI 1640. Once the cells reached 60–70% density, miR-193a-3p mimics or a negative control were co-transfected using Lipofectamine 3000 together with a pmirGLO vector harboring a region of the 3′ end of the untranslated region (3′-UTR) of EFNB2 that contained either a wildtype or mutant version of the putative miR-193a-3p binding site (GenePharma, concentration was 0.5 g/L).

The primary hESCs were extracted as reported (Gellersen et al., 1994) and cultured in Dulbecco modified Eagle medium/Hams F-12 (DMEM/F-12; cat. no. 10-092-CVRC; Corning, Wuhan, China) with 10% FBS (termed complete F12). When the cell confluency reached 80–90%, the complete F12 medium was changed to serum-free medium. After 24 h, the conditioned medium was collected and centrifuged.

### Quantitative Reverse Transcriptase Polymerase Chain Reaction

Total RNA from placental tissues and cultured cells was extracted using RNAiso Plus (cat. no. 9108; TaKaRa, Beijing, China) according to the manufacturer's protocol. For miRNA expression, reverse transcription was performed by using a Mir-X miRNA First-Strand Synthesis Kit (cat. no. 638315; TaKaRa), whereas the PrimeScript RT Reagent Kit with gDNA Eraser (cat. no. RR047A; TaKaRa) was used for mRNA. Then, quantification of miRNAs and mRNAs was completed using TB Green® Premix Ex TaqTM II (Tli RNaseH Plus; cat. no. RR820A; TaKaRa). The U6 gene was applied as an endogenous control for miRNA normalization and GAPDH was used for mRNA normalization. Results were analyzed using the ΔΔCt method. Primer sequences for the qRT-PCR analysis are presented in Table 1.

**Table 1:** Primer sequences for qRT-PCR analysis.

**Gene**	**Forward Premier**	**Reverse Premier**
EFNB2	TAAAGATCCAACAAGACGTCCA	CGTGATGATGATGACGATGAAG
GAPDH	ACAACTTTGGTATCGTGGAAGG	GCCATCACGCCACAGTTTC
miR-193a-3p	cgaAACTGGCCTACAAAGTCCCAGT	
U6	CTCGCTTCGGCAGCACA	AACGCTTCACGAATTTGCGT

### Western Blot

Placental tissues and cultured cells were lysed by radioimmunoprecipitation assay buffer solution (RIPA; cat. no. P0013B; Beyotime, Shanghai, China) with 1% phenylmethanesulfonyl fluoride (PMSF; cat. no. ST506; Beyotime) and were then centrifuged at 12,000 rpm for 20 min at 4°C to collect the supernatant. Protein concentrations were quantified using a Pierce® BCA Protein Assay Kit—Reducing Agent Compatible (cat. no. 23225; Thermo Scientific, Waltham, USA). Samples of equal amounts were separated on 10% SDS-PAGE (cat. no. P0012A; Beyotime) and transferred to polyvinylidene difluoride membrane (cat. no. IPVH00010; Millipore, Darmstadt, Germany). After being blocked with 5% non-fat milk in TBST containing 0.1% Tween 20 for 2 h, the membranes were incubated with primary antibodies overnight at 4°C. The following primary antibodies were used: EFNB2 (1:1,000; cat. no. ab150411; Abcam, Cambridge, UK), E-cadherin (1:1,000; cat. no. 20874-1-AP; Proteintech, Wuhan, China), N-cadherin (1:1,000; cat. no. 22018-1-AP; Proteintech), vimentin (1:1,000; cat. no. 5741; Cell Signaling Technology, Danvers, USA), MMP2 (1:1,000; cat. no. 40994; Cell Signaling Technology), and MMP9 (1:1,000; cat. no. 27306-1-AP; Proteintech). Next, HRP-conjugated secondary antibodies (1:4,000; cat. no. SA00001-1 for anti-mouse and cat. no. SA00001-2 for anti-rabbit; Proteintech) were incubated with the membranes for 90 min and the proteins were detected using Immobilon Western HRP Substrate (cat. no. WBKLS0500; Millipore). GAPDH (1:4,000; cat. no. 60004-1-Ig; Proteintech) was used as an internal control.

### Transwell Migration and Invasion Assay

For the migration assay, a 100-μl cell suspension at a density of 2 × 10^5^ cells/ml with serum-free medium was placed in the upper chamber of a Transwell system (cat. no. 3422; Corning) and the lower chamber was filled with 600 μl hESC-conditioned medium (20% FBS). After a 24-h incubation, the cells were fixed with 4% paraformaldehyde, and crystal violet (cat. no. C0121; Beyotime) was applied for 30 min at room temperature to stain the cells. Five discrete fields of view were selected for counting cell number under the microscope (magnification, ×200) and then averaged.

For invasion assays, the upper chamber of a Transwell system was pre-coated with Matrigel before the cells were plated. Matrigel (cat. no. 354234; Corning), melted overnight at 4°C, was diluted to 1 mg/ml with serum-free medium on ice. Then, 100 μl diluted Matrigel was added into the upper chamber and incubated at 37°C for 5 h. Serum-free medium was added to the Transwells and then the invasion assays were carried out in the same manner as in the migration tests.

### Immunofluorescence Staining

hESCs were seeded on glass coverslips in 12-well-plates at a density of 60–70% and were fixed with 4% paraformaldehyde for 30 min after 24 h. Subsequently, the cells were permeabilized with Triton X-100 (cat. no. P0096; Beyotime) and immediately blocked with 8% bovine serum albumin (cat. no. A600332; BBI Life Sciences, Shanghai, China) with 0.25% Triton X-100 for 1 h at room temperature. After blocking, the cells were incubated with anti-vimentin antibody (1:100) overnight at 4°C. The next day, all processes were performed in the dark. The cells were incubated with secondary antibody (1:200; cat. no. A32740; Invitrogen) for 2 h and 4,6-diamino-2-phenylindole (DAPI; cat. no. C1005; Beyotime) was used to identify nuclei. Images were obtained on a fluorescence microscope.

### Luciferase Assay

HTR-8/SVneo cells were used for a dual-luciferase reporter gene assay. EFNB2 3′-UTR (EFNB2-wt) and mutated EFNB2 3′-UTR (EFNB2-mut) were constructed by GenePharma and luciferase activity was tested using the Dual-Luciferase Reporter Assay System (cat. no. E1910; Promega, Madison, USA) with Renilla luciferase activity as a normalized control.

### Statistical Methods

All continuous data are displayed as the mean ± standard deviation and categorical variables are expressed as a number or percentage. SPSS 24.0 software and GraphPad Prism 7 (GraphPad Software Inc., La Jolla, CA, USA) were used to analyze the statistical data. The Student's *t*-test was used to assess differences in the distribution in continuous data or the Mann–Whitney *U*-test if the data were not normally distributed, whereas the chi-square test or Fisher exact test was used for categorical variables. *P* < 0.05 was considered to indicate a significant difference.

## Results

### miR-193a-3p Is Upregulated in PAS and EFNB2 Is a Direct Target of miR-193a-3p

**Figure 1:**
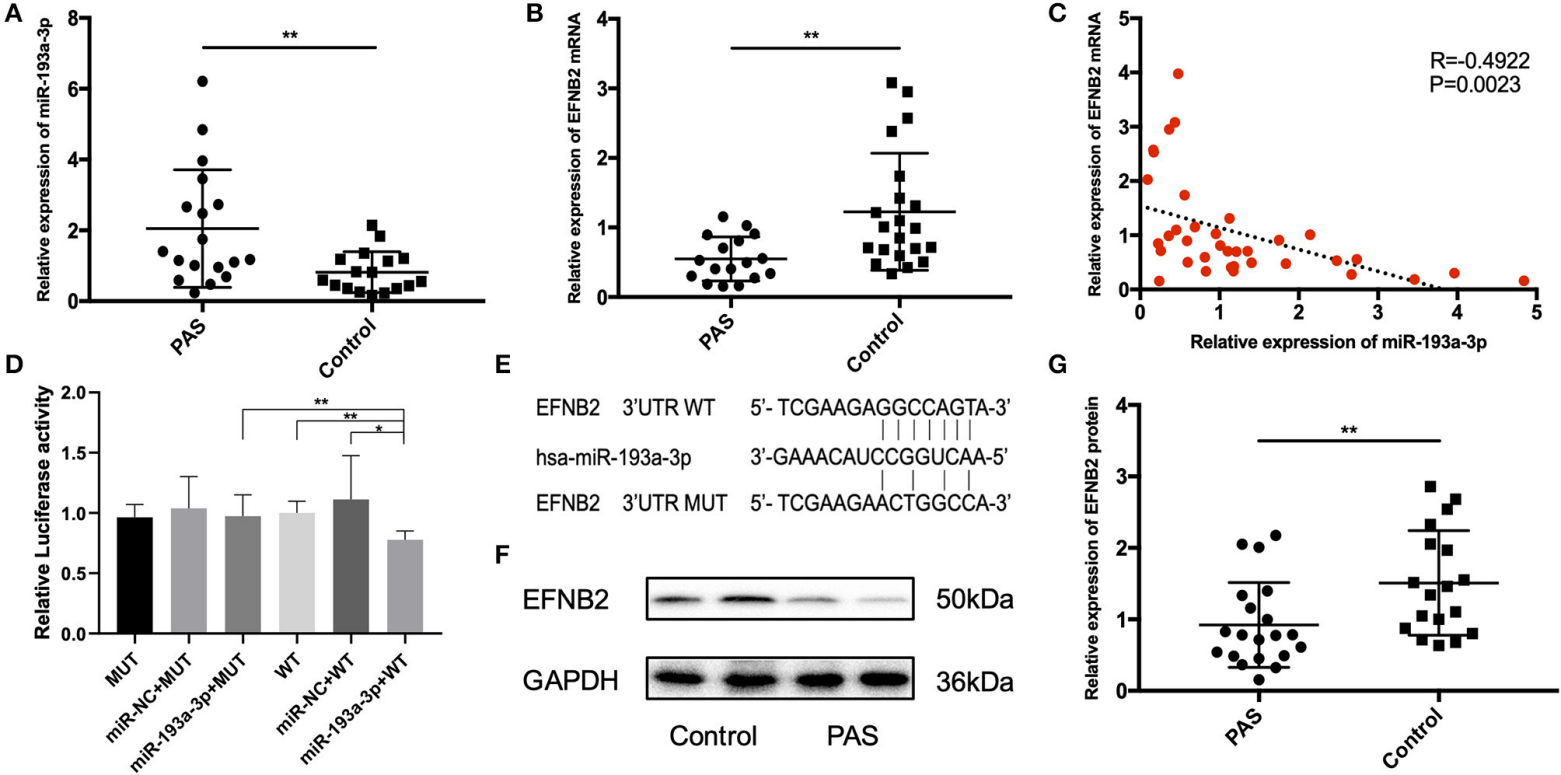
Differential expression of miR-193a-3p and EFNB2 in placenta accreta spectrum tissues. **(A)** miR-193a-3p expression was higher in placental tissues of PAS patients than in those of control patients on qRT-PCR. **(B)** EFNB2 mRNA levels in placental tissues. **(C)** A negative relationship was evident between miR-193a-3p and EFNB2 mRNA in placental tissue samples. **(D)** Relative luciferase activities determined in cells co-transfected with WT or MUT EFNB2 3′-UTR and control, miR-NC, or miR-193a-3p. **(E)** The binding site of miR-193a-3p with WT or MUT EFNB2 3′-UTR. **(F)** Differential expression of EFNB2 protein examined in placental tissues, showing that the expression level of EFNB2 was significantly lower in PAS than in control. **(G)** The gray value measurement method quantitatively analyzes the expression difference of EFNB2/GAPDH in each group. All data are presented as the mean ± standard deviation. PAS, placenta accreta spectrum; EFNB2, Ephrin-B2; miR, microRNA; UTR, untranslated region; WT, wild type; MUT, mutant type; NC, negative control. *P < 0.05, **P < 0.01.

The clinical information of all patients included in the study is shown in Table 2. No variables were significantly different between the two groups. Compared with the control group, the expression of miR-193a-3p was significantly higher in the placental tissues of the PAS group (*P* < 0.05; Figure 1A). In contrast, expression of EFNB2 mRNA was significantly decreased in the placental tissues of the PAS group (*P* < 0.05; Figure 1B) and there was a negative correlation between them (Figure 1C). To investigate whether EFNB2 was directly targeted or suppressed by miR-193a-3p, luciferase reporter assay was performed. The results showed that the fluorescence activity of the wildtype plasmid co-transfected with miR-193a-3p mimics was lower than that of the mutant, indicating that miR-193a-3p targets EFNB2 (Figures 1D,E). Moreover, the EFNB2 protein expression was consistent with the trend in EFNB2 mRNA expression (Figures 1F,G).

**Table 2:** Demographic and obstetrical characteristics.

	**Placenta accreta spectrum**	**Control**	***P*-value**
	***n* = 20**	***n* = 20**	
Age (years)	33.05 ± 4.50	32.20 ± 5.06	0.578
Gestational weeks	36.44 ± 0.75	36.37 ± 0.72	0.760
BMI	28.09 ± 3.59	27.79 ± 3.18	0.787

*Values are presented as the mean ± standard deviation. BMI, body mass index*.

### miR-193a-3p Promotes the Migration and Invasion of HTR-8/SVneo Cells

**Figure 2:**
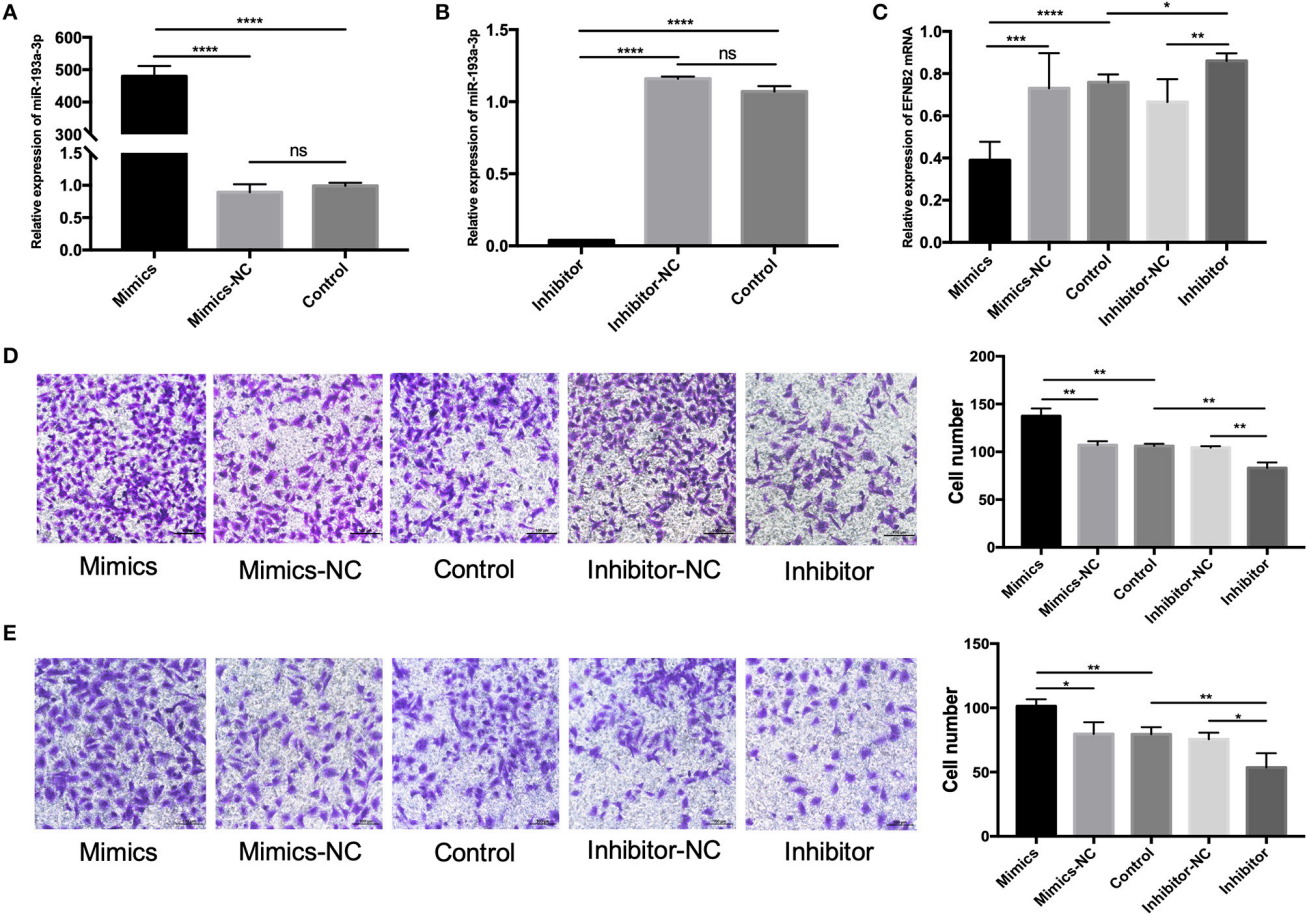
miR-193a-3p promotes the migration and invasion of HTR-8/SVneo cells. **(A,B)** The level of miR-193a-3p increased and decreased after 48-h transfection of HTR-8/SVneo cells with miR-193-3p mimics or miR-193a-3p-inhibitor. **(C)** EFNB2 mRNA expression observed after 48-h transfection with miR-193a-3p-inhibitor or miR-193-3p mimics to demonstrate the relationship between miR-193a-3p and EFNB2. **(D)** Transwell migration assay showing significantly more migrated cells in the mimics group than in the NC group, whereas the migrated cells in the inhibitor group showed the opposite trend (×200). **(E)** Transwell invasion assay revealed more invaded cells in the mimics group than in the NC group, whereas the invaded cells in the inhibitor group showed the opposite trend (×200). All data are presented as the mean ± standard deviation. EFNB2, Ephrin-B2; miR, microRNA; mimics, miR-193a-3p mimics; inhibitor, miR-193a-3p inhibitor; NC, negative control. *P < 0.05, **P < 0.01, ***P < 0.001, and ****P < 0.0001.

To clarify the function of miR-193a-3p, miR-193a-3p mimics and inhibitor were transfected into HTR-8/SVneo cell lines. Primary hESCs were examined due to the major role of decidual defect in the development of PAS. HTR-8/SVneo cells were cultured in complete hESC-conditioned medium after transfection. The hESCs were identified by cell morphology (Supplementary Figure 1A) and immunofluorescence staining with vimentin (a mesenchymal marker) (Supplementary Figure 1B). After transfection with miR-193a-3p mimics and inhibitor for 48 h, the expression level of miR-193a-3p confirmed that the transfection was effective (Figures 2A,B). To further prove that miR-193a-3p directly targets EFNB2, the EFNB2 mRNA was tested after transfection for 48 h (Figure 2C). The results showed a decrease in EFNB2. Transwell experiments performed to explore the function of miR-193a-3p showed that miR-193a-3p mimics significantly increased the migration and invasion rate of HTR-8/SVneo cells but that miR-193a-3p inhibitor had the opposite effect (Figures 2D,E).

### miR-193a-3p Promotes the EMT Pathway in PAS

**Figure 3:**
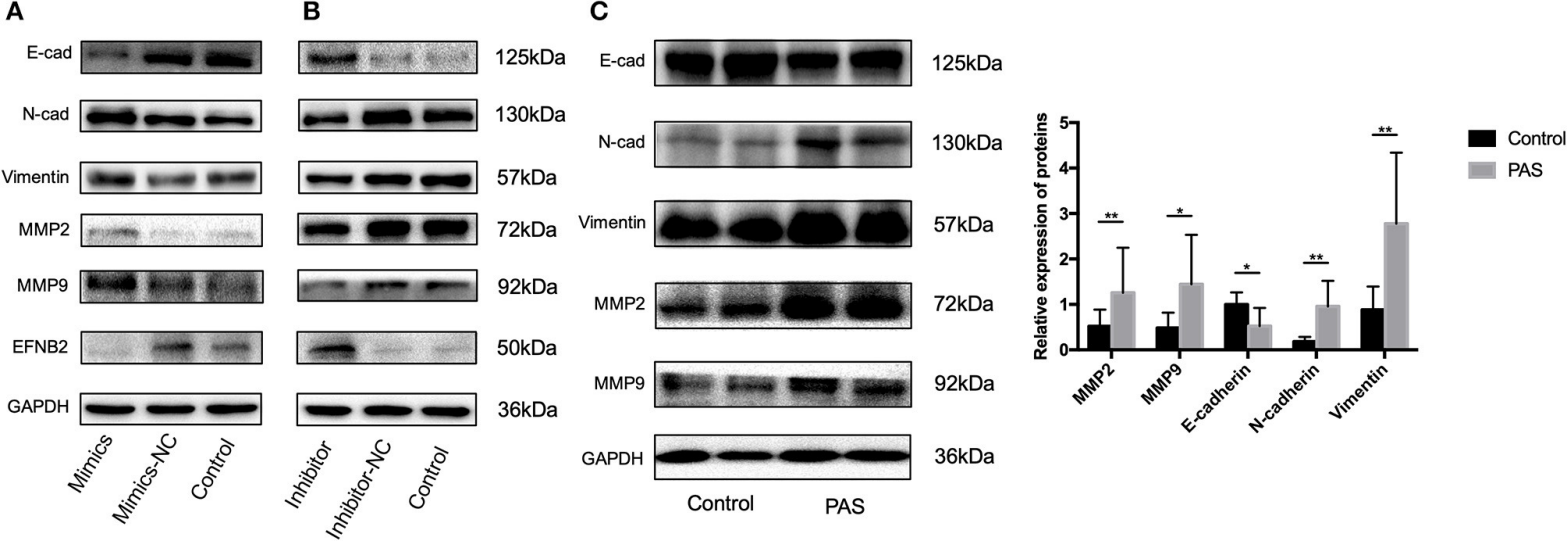
The EMT pathway is activated by miR-193a-3p in placenta accreta spectrum patients. **(A)** Protein levels of E-cad, N-cad, vimentin, MMP2, MMP9, and EFNB2 examined by western blot in HTR-8/SVneo cells transfected with miR-NC and miR-193a-3p mimics. **(B)** Protein levels of E-cad, N-cad, vimentin, MMP2, MMP9, and EFNB2 examined by western blot in HTR-8/SVneo cells transfected with miR-193a-3p-inhibitor-NC and miR-193a-3p-inhibitor. **(C)** Protein levels of E-cad, N-cad, vimentin, MMP2, and MMP9 examined by western blot in placenta accreta spectrum and control placental tissues. All data are presented as the mean ± standard deviation. PAS, placenta accreta spectrum; EFNB2, Ephrin-B2; mimics, miR-193a-3p mimics; inhibitor, miR-193a-3p inhibitor; NC, negative control. *P < 0.05, **P < 0.01.

To elucidate the mechanism underlying the effect of miR-193a-3p on HTR-8/SVneo cell migration and invasion, we investigated the expression of EMT markers and matrix metalloproteinases (MMPs) after transfection with miR-193a-3p for 48 h. The expression levels of N-cadherin, vimentin, MMP2, and MMP9 were increased after miR-193a-3p mimics transfection (Figure 3A). However, E-cadherin was only increased after miR-193a-3p inhibitor transfection (Figure 3B). In addition, the expression level of EFNB2 was also examined, with the results showing that miR-193a-3p decreased the expression of EFNB2. To confirm that these proteins indeed played a critical role in PAS, we compared the placental tissue levels of these proteins between the PAS and control groups. E-cadherin expression was downregulated but N-cadherin, vimentin, MMP2, and MMP9 were upregulated (Figure 3C), indicating that the EMT pathway participates in PAS.

### si-EFNB2 Simulates the Function of miR-193a-3p Mimics in HTR-8/SVneo Cells

**Figure 4:**
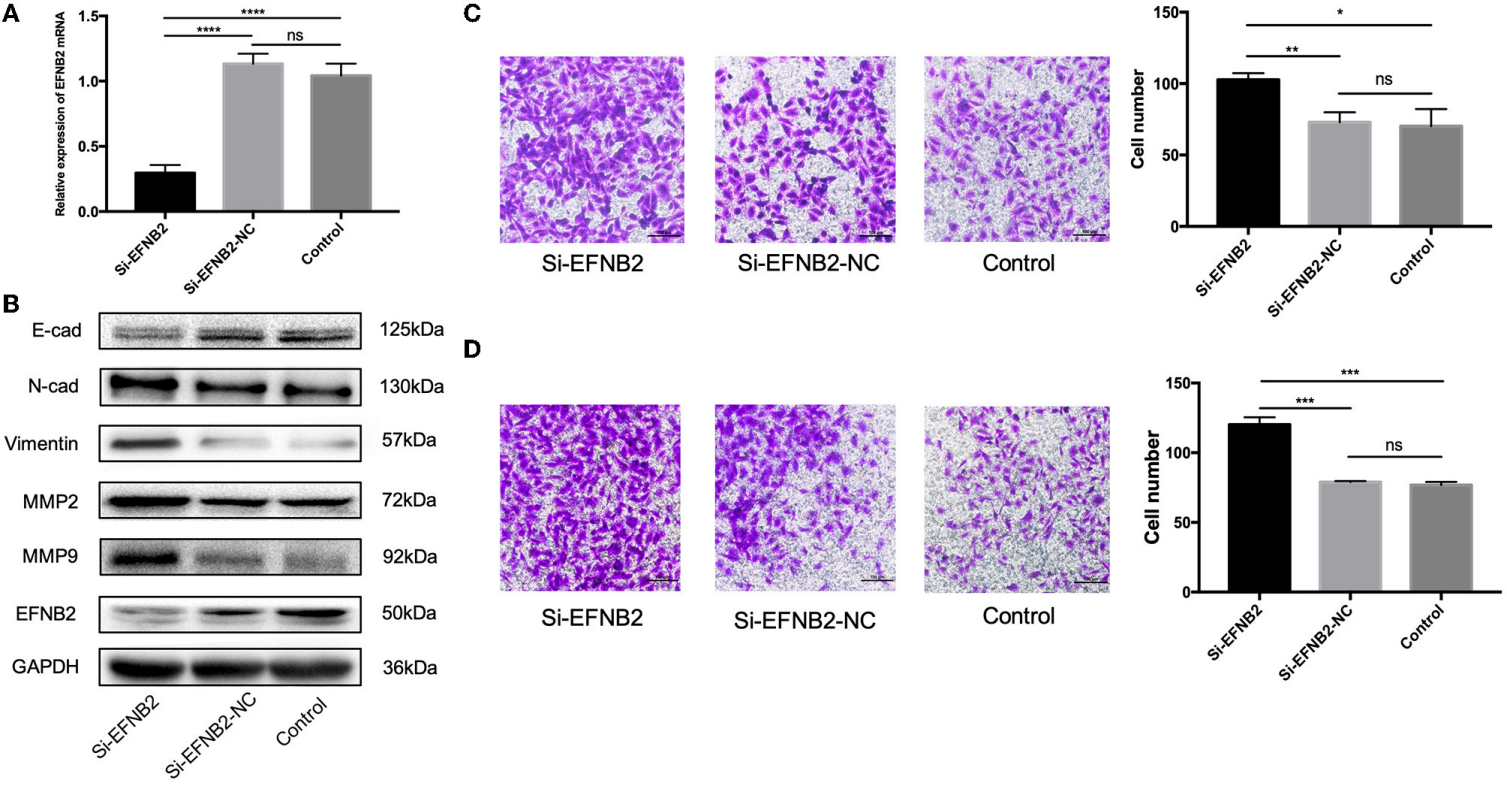
EFNB2 downregulation promotes the migration and invasion of HTR-8/SVneo cells. **(A)** EFNB2 mRNA expression examined after transfection of HTR-8/SVneo cells with siRNA for EFNB2. **(B)** Levels of EFNB2 and EMT-related proteins detected after 48-h transfection. **(C,D)** Transwell migration and invasion assays showing higher numbers of migrated and invaded cells in the si-EFNB2 group than in the NC group (×200). All data are presented as the mean ± standard deviation. EFNB2, Ephrin-B2; Si, siRNA; NC, negative control; EMT, epithelial-mesenchymal transition. *P < 0.05, **P < 0.01, ***P < 0.001, and ****P < 0.0001.

To demonstrate that EFNB2 regulates HTR-8/SVneo cells, similar to miR-193a-3p, the cell line was transfected with si-EFNB2. After 48 h, the mRNA expression levels of EFNB2 indicated 73.96% interference efficiency (Figure 4A). Additionally, si-EFNB2 resulted in increased protein expression of N-cadherin, vimentin, MMP2, and MMP9 and decreased protein expression of EFNB2 and E-cadherin (Figure 4B). Furthermore, functional studies showed increased migration and invasion rates (Figures 4C,D), which were consistent with the trend for miR-193a-3p mimics.

### EFNB2 Downregulation Restores the Ability of miR-193a-3p Inhibitor to Regulate HTR-8/SVneo Cell Migration and Invasion

**Figure 5:**
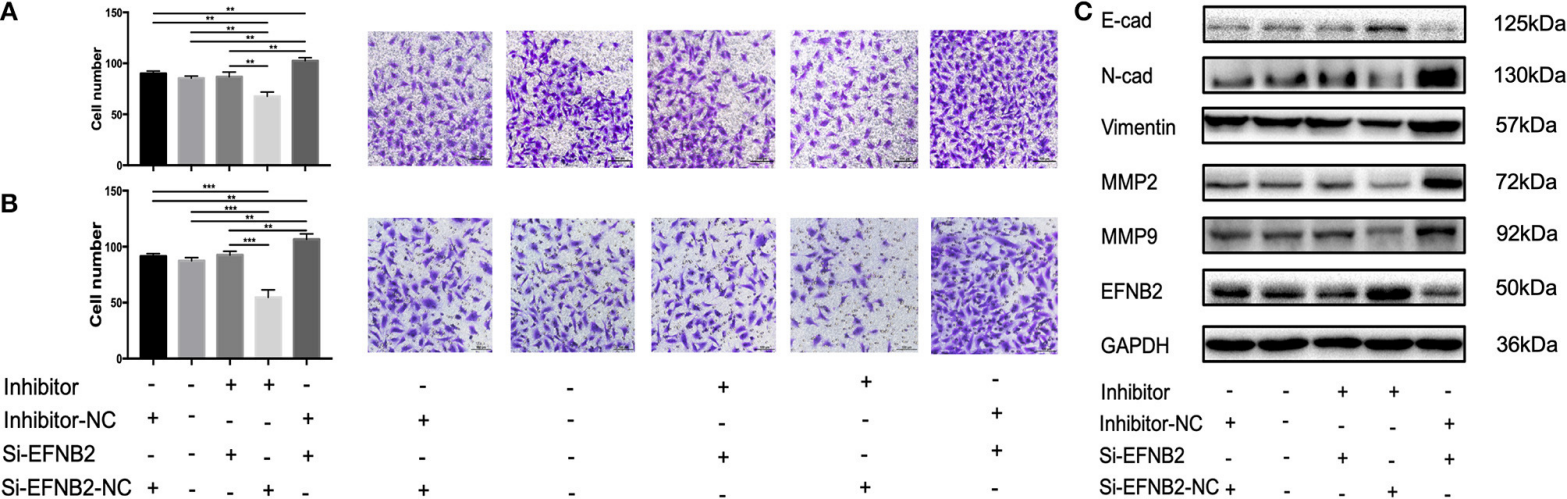
miR-193a-3p regulates the migration and invasion abilities of HTR-8/SVneo cell lines by targeting EFNB2 via the EMT pathway. Migration and invasion abilities of HTR-8/SVneo cells transfected with miR-193a-3p-inhibitor, miR-193a-3p-inhibitor-NC, si-EFNB2, or si-EFNB2-NC examined by Transwell migration assay **(A)** and Transwell invasion assay **(B)**. **(C)** After transfection, the levels of EFNB2 and EMT-related proteins were detected. All data are presented as the mean ± standard deviation. EFNB2, Ephrin-B2; inhibitor, miR-193a-3p inhibitor; Si, siRNA; NC, negative control; EMT, epithelial-mesenchymal transition. **P < 0.01 and ***P < 0.001.

To explore whether the effects of miR-193a-3p were EFNB2 dependent, HTR-8/SVneo cells were transfected with si-EFNB2-NC (negative control) + miR-193a-3p inhibitor, si-EFNB2 + miR-193a-3p inhibitor, si-EFNB2 + miR-193a-3p inhibitor-NC, or si-EFNB2-NC + miR-193a-3p inhibitor-NC. After co-transfection, Transwell experiments revealed that downregulation of EFNB2 partly restored the negative effects of miR-193a-3p inhibitor on cell migration and invasion activity (Figures 5A,B). Moreover, western blotting showed that si-EFNB2 significantly reduced the expression of EFNB2 and E-cadherin induced by miR-193a-3p inhibitor. Analogously, the expression levels of N-cadherin, vimentin, MMP2, and MMP9 were rescued, with an increase in the si-EFNB2 + miR-193a-3p inhibitor group (Figure 5C).

## Discussion

During pregnancy, ESCs undergo decidualization and become decidual cells when the embryo implants into the uterus. At the same time, the decidua can be divided into three parts—the decidua basalis, the decidua parietalis, and the decidua capsularis—based on the point of contact with fetal tissues (Cunningham et al., 2014). Communication between the decidual tissue, particularly the decidua basalis, and the trophoblast is critical for pregnancy and undifferentiated ESCs can promote trophoblast invasiveness (Wongwananuruk et al., 2016). In our study, due to the hypothesis that decidua defects are the primary cause of PAS, we established a cell model based on complete hESC-conditioned medium, which provided a source of growth factors and cytokines and which stimulated the microenvironment between trophoblasts and the defect decidua *in vitro*.

miRNAs play major roles in the regulation of genes involved in diverse biological processes, including embryo implantation and placental development (Liang et al., 2017; Hayder et al., 2018). The placenta is a transient organ designed to support the fetus and make it possible for the fetus to communicate with the mother. Therefore, a number of obstetric diseases can occur if the placenta abnormally develops, such as preeclampsia, fetal growth restriction, and gestational diabetes mellitus (Higashijima et al., 2013; Cai et al., 2017; Gunel et al., 2017; Hosseini et al., 2018). However, it is largely unknown how miRNAs regulate the function of the placenta in PAS. In previous studies, miR-34a, miR-29a/b/c and miR-125a were identified in PAS patients (Umemura et al., 2013; Gu et al., 2016, 2019). Our work revealed miR-193a-3p overexpression in the placental tissue in PAS patients. The four miRNAs might regulate different or same mRNAs to promote the development of PAS because there is a certain compensation effect between functionally related miRNAs. It is generally believed that the processing and production of each miRNA in a miRNA cluster is independently carried out and whether different miRNAs clusters affect each other is unknown, resulting in more research about these four miRNAs relationships in the future. In the physiological environment, miR-193a-3p can limit the proliferation and cell cycle progression of normal cells, and can play a tumor suppressor effect in cancer by targeting different genes related to proliferation, apoptosis, migration, invasion and metastasis (Nakano et al., 2013; Liang et al., 2015; Williams et al., 2015; Grossi et al., 2017; Khoo et al., 2017). However, several studies have reported that miR-193a-3p can serve as an oncogene in different cancers to promote cell migration (Yi et al., 2016; Liu et al., 2017; Pan et al., 2018), which is in accordance with our results showing that miR-193a-3p boosted trophoblast cell migration and invasion. The results might be related to the microenvironment of maternal-fetal interface (complete hESC-conditioned medium without decidua) and the genes regulated by miR-193a-3p in this microenvironment, which leads to invasive trophoblasts penetrate hESC, enter myometrium, and cause PAS.

PAS is a process of tissue migration that might involve EMT (DaSilva-Arnold et al., 2018). Regulation of EMT has been shown to occur at the level of epigenetics and miRNA (Loffredo et al., 2017). Although Umemura et al. (2013) found that the expression of miR-34a was downregulated in PAS without functional studies, many studies have reported that miR-34a inhibits EMT by repressing several key molecules (Huang et al., 2018). The mechanism that miR-193a-3p regulates the EMT pathway may be achieved by directly regulating the related proteins of the EMT pathway, or may be mediated by other target genes. Liu et al. (2017) determined that miR-193a-3p enhanced cell growth and migration by targeting PTEN. However, Pan et al. (2018) found that the gene targeted by miR-193a-3p—alpha-2,3-sialyltransferase IV (ST3GalIV)—promoted cell migration and invasion in renal cell carcinoma. In this study, we evaluated the function of miR-193a-3p and identified EFNB2 as a critical target gene, which was proved by our luciferase assay results. Furthermore, EFNB2 was decreased in the PAS group and suppressed trophoblast migration and invasion. Additionally, previous work reported roles for Eph receptors in EMT (Li et al., 2014; Loffredo et al., 2017). Therefore, EMT-related proteins were examined to clarify the mechanism underlying the ability of miR-193a-3p to target EFNB2 in the present work. We found that miR-193a-3p mimics and si-EFNB2 increased the expression level of N-cadherin, vimentin, MMP2, and MMP9 and decreased the expression level of E-cadherin. The placental tissues from patients with PAS also showed the same results. Therefore, we thought miR-193a-3p can induce EMT by negatively regulating EFNB2.

However, how the EFNB2 induces EMT was unknown. EFNB2 repression induced tumor migration and invasion in human gliomas and breast cancer, which was similar to our results (Depner et al., 2016; Magic et al., 2019). In contrast, prior work determined that a decrease in EFNB2 levels impaired the migration/invasion capabilities of HTR-8/SVneo cells (Luo et al., 2015). We have to emphasize that our cells were cultured in complete hESC-conditioned medium, which might have influenced the secretion of several cytokines and alter how EFNB2 affects the HTR-8/SVneo cells. Moreover, EFNB2/EPHB4 bidirectional signaling is complex during disease development and how EFNB2 plays a role in PAS remains uncertain, indicating that further work is required to determine whether EFNB2 promotes or suppresses trophoblast migration/invasion.

Nevertheless, there are several limitations to the present study. First, although we established a cell model to mimic the trophoblast and decidua microenvironment due to a lack of animal models, the experiments were mostly *in vitro* and based on a human trophoblast cell line. What's more, several studies have brought into question the true nature of HTR-8/SVneo cell line (Msheik et al., 2020). Thus, the mechanism underlying the role of miR-193a-3p in PAS still requires further studies in other cell lines and *in vivo* research, possible if an animal model is developed. Second, the samples were all from term placental tissues, even though trophoblast invasion occurs in the early trimester. Our results would be more meaningful if we could explore the roles of miR-193a-3p from the early pregnancy to term. Third, the expression of miR-193a-3p should be detected in the peripheral blood of patients with PAS, in order to examine there is the possibility of using miR-193a-3p levels to prevent and monitor PAS. Fourth, the placental functions at the biochemical level in response to miR-193a-3p on EFBN2 expression needs to verify. Finally, EFNB2, an arterial marker, should be further studied in the angiogenesis-related pathways in PAS, as well as miR-193a-3p.

In conclusion, we have determined that miR-193a-3p is upregulated in the placental tissues of patients with PAS and that miR-193a-3p can promote trophoblast migration and invasion that depends on EFNB2 and the EMT pathway under decidua defect conditions.

## Data Availability Statement

The original contributions presented in the study are included in the article/Supplementary Material, further inquiries can be directed to the corresponding author/s.

## Ethics Statement

The study was approved by Shengjing Hospital and China Medical University. The patients/participants provided their written informed consent to participate in this study.

## Author Contributions

CL and JW proposed the concept and supervised the study. RH and TY helped with the experimentation and data analysis. NL was in charge of the literature search, experiments, data analysis, and writing of the article. All authors contributed to the article and approved the submitted version.

## Conflict of Interest

The authors declare that the research was conducted in the absence of any commercial or financial relationships that could be construed as a potential conflict of interest.

## Supplementary Material

Supplementary Figure 1 Identification of primary hESCs. **(A)** Cell morphologies of primary hESCs. (i) indicates ×100 and (ii) indicates ×200. **(B)** Immunofluorescence staining with anti-vimentin antibody. Red indicates vimentin; blue indicates nuclei stained with DAPI. hESC, human endometrial stromal cell. DAPI, 4,6-diamino-2-phenylindole.
