# Corrigendum: miR-193a-3p Mediates Placenta Accreta Spectrum Development by Targeting EFNB2 via Epithelial-Mesenchymal Transition Pathway Under Decidua Defect Conditions

**DOI:** 10.3389/fmolb.2022.839235

**Published:** 2022-02-15

**Authors:** Na Li, Rui Hou, Tian Yang, Caixia Liu, Jun Wei

**Affiliations:** ^1^ Department of Obstetrics and Gynecology, Shengjing Hospital of China Medical University, Shenyang, China; ^2^ Key Laboratory of Maternal-Fetal Medicine of Liaoning Province, Key Laboratory of Obstetrics and Gynecology of Higher Education of Liaoning Province, Benxi, China

**Keywords:** miR-193a-3p, EFNB2, epithelial-mesenchymal transition, decidua defect, placenta accreta spectrum

In the original article, there was a mistake in Figure 4 as published. The picture of “Control” in Figure 4C was wrong when we upload the pictures. The corrected Figure 4 appears below.

**FIGURE 4:**
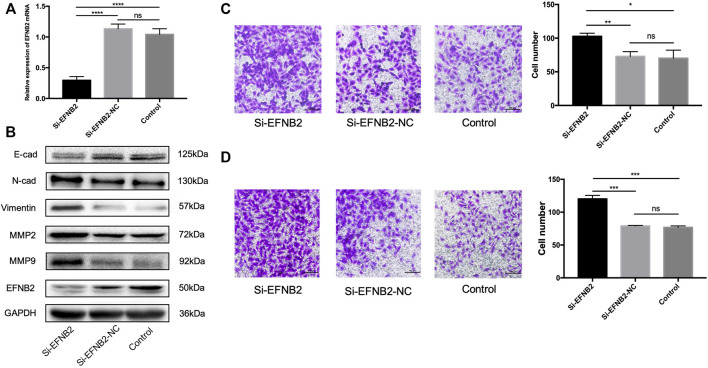
EFNB2 downregulation promotes the migration and invasion of HTR-8/SVneo cells. **(A)** EFNB2 mRNA expression examined after transfection of HTR-8/SVneo cells with siRNA for EFNB2. **(B)** Levels of EFNB2 and EMT-related proteins detected after 48-h transfection. **(C,D)** Transwell migration and invasion assays showing higher numbers of migrated and invaded cells in the si-EFNB2 group than in the NC group (×200). All data are presented as the mean ± standard deviation. EFNB2, Ephrin-B2; Si, siRNA; NC, negative control; EMT, epithelial-mesenchymal transition. *P < 0.05, **P < 0.01, ***P < 0.001, and ****P < 0.0001.

The authors apologize for this error and state that this does not change the scientific conclusions of the article in any way. The original article has been updated.

