# Supplementary figures and images for: miR-193a-3p Mediates Placenta Accreta Spectrum Development by Targeting EFNB2 via Epithelial-Mesenchymal Transition Pathway Under Decidua Defect Conditions
Source: Front Mol Biosci. 2021 Jan 13;7:613802. doi: 10.3389/fmolb.2020.613802 (PMC7873918; doi:10.3389/fmolb.2020.613802)

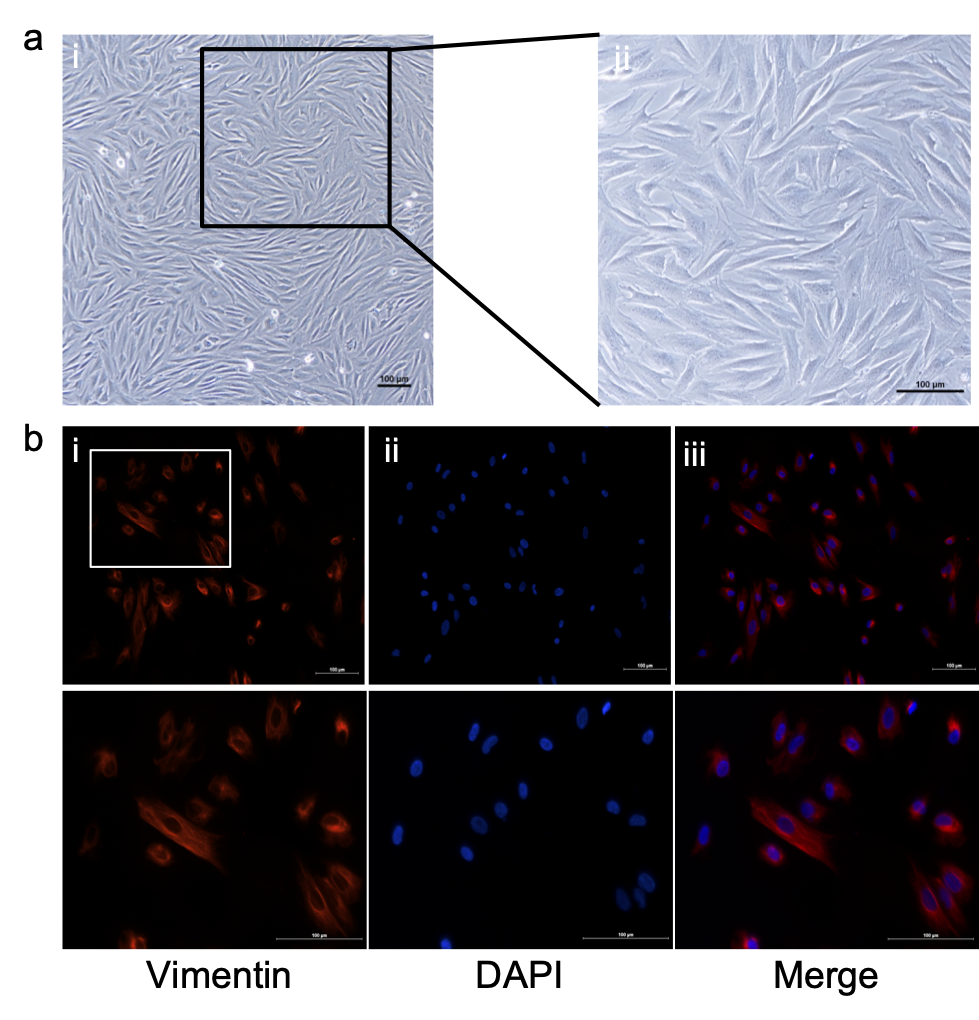

Supplement: Supplementary Figure 1 — Identification of primary hESCs. (A) Cell morphologies of primary hESCs. (i) indicates ×100 and (ii) indicates ×200. (B) Immunofluorescence staining with anti-vimentin antibody. Red indicates vimentin; blue indicates nuclei stained with DAPI. hESC, human endometrial stromal cell. DAPI, 4,6-diamino-2-phenylindole. [file Image_1.TIFF]
