# Supplementary material for: miR-193a-3p Mediates Placenta Accreta Spectrum Development by Targeting EFNB2 via Epithelial-Mesenchymal Transition Pathway Under Decidua Defect Conditions
Source: Front Mol Biosci. 2021 Jan 13;7:613802. doi: 10.3389/fmolb.2020.613802 (PMC7873918; doi:10.3389/fmolb.2020.613802)
Supplement: Supplementary file 2 [file Data_Sheet_1.PDF]

Certificate of Analysis

pmirGLO Dual-Luciferase miRNA Target Expression Vector:

Cat.#	Size
E1330	20µg

Part# 9PIE133
Revised 10/09

Cat.# E1330 contains:

Part No.	Name	
E133A	pmirGLO Vector	20µg
C838A	Oligo Annealing Buffer	1ml

Description: The pmirGLO Dual-Luciferase miRNA Target Expression Vector[(a–e)] is designed to quantitatively evaluate microRNA (miRNA) activity by the insertion of miRNA target sites 3´ of the firefly luciferase gene (*luc2*). These target sites can be introduced by cloning putative miRNA binding sites alone, or the 3´ untranslated region (UTR) of a gene of interest, to study the influence of these sites on transcript stability and activity. Firefly luciferase is the primary reporter gene; reduced firefly luciferase expression indicates the binding of endogenous or introduced miRNAs to the cloned miRNA target sequence. This vector is based on Promega dual-luciferase technology, with firefly luciferase (*luc2*) used as the primary reporter to monitor mRNA regulation and *Renilla* luciferase (*hRluc-neo*) acting as a control reporter for normalization and selection. This vector contains the following features:

- Human phosphoglycerate kinase (PGK) promoter provides low translational expression, which is advantageous when reduction of signal is the desired response. The PGK promoter is a nonviral universal promoter, which functions across cell lines (yeast, rat, mouse and human).
- Firefly luciferase reporter gene (*luc2*) inversely reports miRNA activity in mammalian cells.
- Multiple cloning site (MCS) is located 3´ of the firefly luciferase reporter gene (*luc2*).
- Humanized *Renilla* luciferase-neomycin resistance cassette (*hRluc*-neo) is used as a control reporter for normalization of gene expression and stable cell line selection.
- Amp^r gene allows bacterial selection for vector amplification.
- SV40 late poly(A) signal sequence is positioned downstream of *luc2* to provide efficient transcription termination and mRNA polyadenylation.
- Synthetic poly(A) signal/transcription stop site.

Concentration: 1µg/µl in 10mM Tris-HCl, 1mM EDTA; final pH 7.4.

GenBank® Accession Number: FJ376737.

Storage Conditions: See the storage temperature and expiration date on the Product Information Label.

AF9PI E133 1009E133

Promega Corporation
2800 Woods Hollow Road
Madison, WI 53711-5399 USA
Telephone 608-274-4330
Toll Free 800-356-9526
Fax 608-277-2516
Internet www.promega.com

Quality Control Assays

Functional Assays

Identity Assay: The vector has been sequenced completely and has 100% identity with the published sequence available at: **www.promega.com/vectors/**

Restriction Digestion: The functional purity of this vector DNA is verified by complete digestion with restriction enzymes at the optimal temperature for 1 hour. Samples are examined by agarose gel electrophoresis, comparing cut and uncut vector DNA with marker DNA.

Contaminant Assays

Contaminating Nucleic Acids: RNA, single-stranded DNA and chromosomal DNA are not evident in specified quantities of this vector as determined by agarose gel electrophoresis.

Nuclease Assay: Following incubation of 1µg of this vector in Restriction Enzyme Buffer at 37°C for 16–24 hours, no evidence of nuclease activity is detected by agarose gel electrophoresis.

Physical Purity: $A_{260}/A_{280} \geq 1.80$, $A_{260}/A_{250} \geq 1.05$.

Signed by: J. Stevens

J. Stevens, Quality Assurance

[(a)]READ THIS FIRST BEFORE OPENING PRODUCT
The sale of this product and its use by the purchaser are subject to the terms of a limited use label license, the full text of which is shipped with this product and also available at: **www.promega.com\LULL**. That text must be read by the purchaser prior to opening this product to determine whether the purchaser agrees that all use of the product shall be in accordance with the license terms. If the purchaser is not willing to accept the terms of the limited use label license, Promega is willing to accept the return of the unopened product and provide the purchaser with a full refund. However, if the product is opened for any reason, then the purchaser agrees to be bound by the terms of the limited use label license.
[(b)]U.S. Pat. No. 5,670,356.
[(c)]Australian Pat. No. 2001 285278 and other patents pending.
[(d)]The method of recombinant expression of *Coleoptera* luciferase is covered by U.S. Pat. Nos. 5,583,024, 5,674,713 and 5,700,673. A license (from Promega for research reagent products and from The Regents of the University of California for all other fields) is needed for any commercial sale of nucleic acid contained within or derived from this product.
[(e)]Licensed from University of Georgia Research Foundation, Inc., under U.S. Pat. Nos. 5,292,658, 5,418,155, Canadian Pat. No. 2,105,984 and related patents.

PRODUCT USE LIMITATIONS, WARRANTY, DISCLAIMER

Promega manufactures products for a number of intended uses. Please refer to the product label for the intended use statements for specific products. Promega products contain chemicals which may be harmful if misused. Due care should be exercised with all Promega products to prevent direct human contact.

Each Promega product is shipped with documentation stating specifications and other technical information. Promega products are warranted to meet or exceed the stated specifications. Promega's sole obligation and the customer's sole remedy is limited to replacement of products free of charge in the event products fail to perform as warranted. Promega makes no other warranty of any kind whatsoever, and SPECIFICALLY DISCLAIMS AND EXCLUDES ALL OTHER WARRANTIES OF ANY KIND OR NATURE WHATSOEVER, DIRECTLY OR INDIRECTLY, EXPRESS OR IMPLIED, INCLUDING, WITHOUT LIMITATION, AS TO THE SUITABILITY, PRODUCTIVITY, DURABILITY, FITNESS FOR A PARTICULAR PURPOSE OR USE, MERCHANTABILITY, CONDITION, OR ANY OTHER MATTER WITH RESPECT TO PROMEGA PRODUCTS. In no event shall Promega be liable for claims for any other damages, whether direct, incidental, foreseeable, consequential, or special (including but not limited to loss of use, revenue or profit), whether based upon warranty, contract, tort (including negligence) or strict liability arising in connection with the sale or the failure of Promega products to perform in accordance with the stated specifications.

© 2008, 2009 Promega Corporation. All Rights Reserved.

Dual-Glo is a registered trademark of Promega Corporation. GeneClip and PureYield are trademarks of Promega Corporation.

GenBank is a registered trademark of US Department of Health and Human Services.

Products may be covered by pending or issued patents or may have certain limitations. Please visit our Web site for more information.

All specifications are subject to change without prior notice.

Product claims are subject to change. Please contact Promega Technical Services or access the Promega online catalog for the most up-to-date information on Promega products.

Part# 9PIE133
Printed in USA Revised 10/09

Features List and Map for the pmirGLO Vector

SV40 late poly(A) signal	106–327
SV40 early enhancer/promotor	426–844
hRluc-neo fusion protein coding region	889–2664
Synthetic polyadenylation signal	2728–2776
β-lactamase (Amp^r) coding region	3037–3897
*Col*E1-derived plasmid origin of replication	4052–4088
Human phosphoglycerate kinase promoter	5094–5609
luc2 reporter gene	5645–7297
Multiple cloning site (MCS, Figure 1)	7306–7350

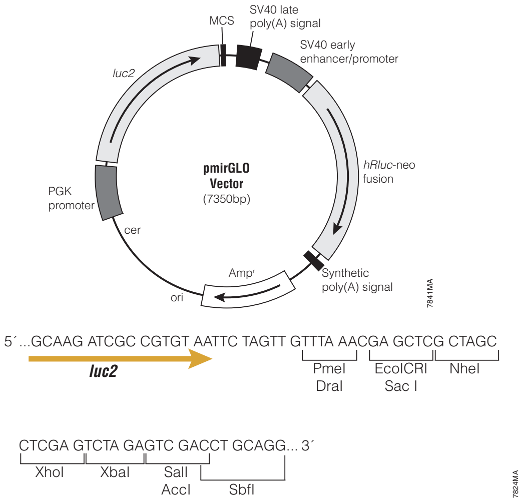

Figure 1. pmirGLO Vector multiple cloning site.

I. Sample Protocol

A. Vector Cloning

1. Design oligonucleotides: Order oligonucleotide pairs that contain the desired miRNA target region and, when annealed and ligated into the pmirGLO Vector, result in the miRNA target region in the correct 5´ to 3´ orientation. Insure that the overhangs created by oligonucleotide annealing are complementary to those generated by restriction enzyme digestion of the pmirGLO Vector in Step 2. Add an internal restriction site to your oligonucleotides for clone confirmation (e.g., NotI in Figure 3 creates a ~125bp insert when digested with NotI because of a NotI site at position 93 in the vector).
2. Digest vector: Linearize the pmirGLO Vector with the appropriate restriction enzymes to generate overhangs that are complementary to the annealed oligonucleotide overhangs.
3. Anneal oligonucleotides: Dilute both oligonucleotides (supplied by user) to 1µg/µl. Combine 2µl of each oligonucleotide with 46µl of Oligo Annealing Buffer. Heat at 90°C for 3 minutes, then transfer to a 37°C water bath for 15 minutes. Use the annealed oligonucleotides immediately, or store at –20°C.

B. Ligation and Transformation

1. Dilute annealed oligonucleotides 1:10 in nuclease-free water to a final concentration of 4ng/µl per oligonucleotide. Ligate 4ng of annealed oligonucleotides and 50ng of linearized vector using a standard ligation protocol. Transform ligated pmirGLO Vector using high-efficiency JM109 competent cells (e.g., Cat.# L2001).
2. Select clones on ampicillin-containing plates, then select clones containing the oligonucleotides by digesting miniprep-purified DNA (e.g., purified using the PureYield™ Plasmid Miniprep System, Cat.# A1221) using the unique restriction site in the oligonucleotide pair. The purified plasmid DNA can be transfected directly or expanded to generate more DNA.

Additional information about annealing, ligation, transformation and oligonucleotide design can be found in the *GeneClip™ U1 Hairpin Cloning Systems Technical Manual*, #TM256, which is available at: **www.promega.com/tbs/**

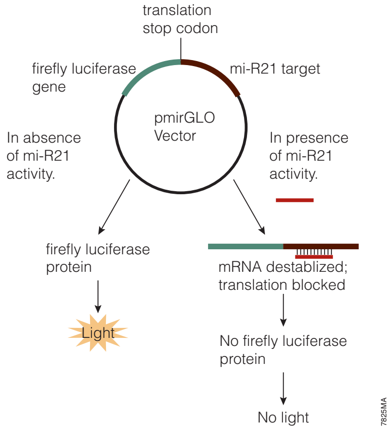

Figure 2. Mechanism of action of the pmirGLO Vector.

	PmeI NotI internal site mi-R21 target sequence XbaI
**mi-R21 sense, PmeI and XbaI**	5´ **AAAC** TA GCGGCCGC TAGT TCAACATCAG*TCT*GATAAGCTA **T** 3´
**mi-R21 mismatch sense, PmeI and XbaI**	5´ **AAAC** TA GCGGCCGC TAGT TCAACATCAG*AA*GATAAGCTA **T** 3´

	XbaI mi-R21 target sequence NotI internal site PmeI
**mi-R21 antisense, PmeI and XbaI**	5´ **CTAGA** TAGCTTATC*AGA*CTGATGTTGA ACTA GCGGCCGC TA **GTTT** 3´
**mi-R21 mismatch antisense, PmeI and XbaI**	5´ **CTAGA** TAGCTTATC*TT*CTGATGTTGA ACTA GCGGCCGC TA **GTTT** 3´

7826MA

Figure 3. Sample oligonucleotides for mi-R21.

C. An Example of Detecting mi-R21 Activity Using the pmirGLO Vector:miR-21 Construct

An overview describing the use of the pmirGLO Vector to interrogate endogenous mi-R21 microRNA is shown in Figure 2.

The presence of broadly endogenous microRNA mi-R21 was monitored in HeLa cells. Constructs contained either an exact match to the 21bp mi-R21 target sequence or a mismatched version of that target site (1) as well as PmeI, XbaI and NotI restriction sites (Figure 3; mismatched sequence is in italics). Twenty-four hours after transfection with the mi-R21 pmirGLO Vector constructs, cells were analyzed for luciferase activity using the Dual-Glo® Luciferase Assay System (Cat.# E2920) and a MicroLumatPlus LB96V luminometer (Berthold). Normalized firefly luciferase activity (firefly luciferase activity/*Renilla* luciferase activity) for each construct was compared to that of the pmirGLO Vector no-insert control. For each transfection, luciferase activity was averaged from six replicates.

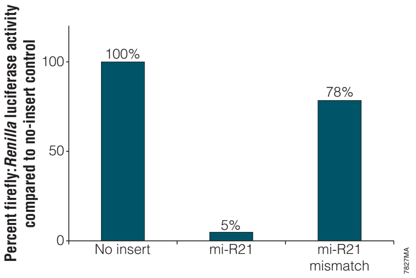

Figure 4. Normalized luciferase activity using the pmirGLO Vector with an mi-R21 target sequence.

II. Reference

1. Zeng, Y. and Cullen, B.R. (2003) Sequence requirements for micro RNA processing and function in human cells. *RNA* **9**, 112–23.

Part# 9PIE133
Printed in USA Revised 10/09
